# Lesão Miocárdica e Prognóstico em Pacientes Hospitalizados com COVID-19 no Brasil: Resultados do Registro Nacional de COVID-19

**DOI:** 10.36660/abc.20220151

**Published:** 2023-02-13

**Authors:** Hannah Cardoso Barbosa, Maria Auxiliadora Parreiras Martins, Jordana Cristina de Jesus, Karina Cardoso Meira, Luiz Guilherme Passaglia, Manuela Furtado Sacioto, Adriana Falangola Benjamin Bezerra, Alexandre Vargas Schwarzbold, Amanda de Oliveira Maurílio, Barbara Lopes Farace, Carla Thais Cândida Alves da Silva, Christiane Corrêa Rodrigues Cimini, Daniel Vitorio Silveira, Daniela do Reis Carazai, Daniela Ponce, Emanuel Victor Alves Costa, Euler Roberto Fernandes Manenti, Evelin Paola de Almeida Cenci, Frederico Bartolazzi, Glícia Cristina de Castro Madeira, Guilherme Fagundes Nascimento, Isabela Vasconcellos Pires Velloso, Joanna d’Arc Lyra Batista, Júlia Drumond Parreiras de Morais, Juliana da Silva Nogueira Carvalho, Karen Brasil Ruschel, Karina Paula Medeiros Prado Martins, Liege Barella Zandoná, Luanna Silva Monteiro Menezes, Luciane Kopittke, Luís César de Castro, Luiz Antônio Nasi, Maiara Anschau Floriani, Maíra Dias Souza, Marcelo Carneiro, Maria Aparecida Camargos Bicalho, Maria Clara Pontello Barbosa Lima, Mariana Frizzo de Godoy, Milton Henriques Guimarães-Júnior, Paulo Mascarenhas Mendes, Polianna Delfino-Pereira, Raquel Jaqueline Eder Ribeiro, Renan Goulart Finger, Rochele Mosmann Menezes, Saionara Cristina Francisco, Silvia Ferreira Araújo, Talita Fischer Oliveira, Thainara Conceição de Oliveira, Carisi Anne Polanczyk, Milena Soriano Marcolino

**Affiliations:** 1 Hospital das Clínicas Universidade Federal de Minas Gerais Belo Horizonte MG Brasil Hospital das Clínicas - Universidade Federal de Minas Gerais,Belo Horizonte, MG – Brasil; 2 Universidade Federal do Rio Grande do Norte Natal RN Brasil Universidade Federal do Rio Grande do Norte,Natal, RN – Brasil; 3 Faculdade Ciências Médicas de Minas Gerais Belo Horizonte MG Brasil Faculdade Ciências Médicas de Minas Gerais, Belo Horizonte, MG – Brasil; 4 Hospital das Clínicas Universidade Federal de Pernambuco Recife PE Brasil Hospital das Clínicas da Universidade Federal de Pernambuco, Recife, PE – Brasil; 5 Hospital Universitário de Santa Maria Santa Maria RS Brasil Hospital Universitário de Santa Maria,Santa Maria, RS – Brasil; 6 Hospital São João de Deus Divinópolis MG Brasil Hospital São João de Deus,Divinópolis, MG – Brasil; 7 Hospital Risoleta Tolentino Neves Belo Horizonte MG Brasil Hospital Risoleta Tolentino Neves,Belo Horizonte, MG – Brasil; 8 Hospital Santo Antônio Curvelo MG Brasil Hospital Santo Antônio,Curvelo, MG – Brasil; 9 Hospital Santa Rosália Teófilo Otoni MG Brasil Hospital Santa Rosália,Teófilo Otoni, MG – Brasil; 10 Universidade Federal dos Vales do Jequitinhonha e Mucuri Teófilo Otoni MG Brasil Universidade Federal dos Vales do Jequitinhonha e Mucuri, Teófilo Otoni, MG – Brasil; 11 Hospital UNIMED BH Belo Horizonte MG Brasil Hospital UNIMED BH,Belo Horizonte, MG – Brasil; 12 Hospital Nossa Senhora da Conceição Porto Alegre RS Brasil Hospital Nossa Senhora da Conceição, Cristo Redentor, Porto Alegre, RS – Brasil; 13 Universidade Estadual Paulista Botucatu SP Brasil Universidade Estadual Paulista, Botucatu, SP – Brasil; 14 Centro Universitário de Belo Horizonte Belo Horizonte MG Brasil Centro Universitário de Belo Horizonte (UniBH),Belo Horizonte, MG – Brasil; 15 Hospital Mãe de Deus Porto Alegre RS Brasil Hospital Mãe de Deus,Porto Alegre, RS – Brasil; 16 Hospital Universitário Canoas Canoas RS Brasil Hospital Universitário Canoas,Canoas, RS – Brasil; 17 Hospitais da Rede Mater Dei Belo Horizonte MG Brasil Hospitais da Rede Mater Dei,Belo Horizonte, MG – Brasil; 18 Universidade Federal da Fronteira Sul Chapecó SC Brasil Universidade Federal da Fronteira Sul, Chapecó, SC – Brasil; 19 Hospital Regional do Oeste Chapecó SC Brasil Hospital Regional do Oeste, Chapecó, SC – Brasil; 20 Hospital Universitário Ciências Médicas Belo Horizonte MG Brasil Hospital Universitário Ciências Médicas,Belo Horizonte, MG – Brasil; 21 IATS CNPq Porto Alegre RS Brasil Instituto de Avaliação de Tecnologia em Saúde (IATS/ CNPq),Porto Alegre, RS – Brasil; 22 Hospital Bruno Born Lajeado RS Brasil Hospital Bruno Born,Lajeado, RS – Brasil; 23 Hospital Luxemburgo Belo Horizonte MG Brasil Hospital Luxemburgo, Belo Horizonte, MG – Brasil; 24 Hospital Municipal Odilon Behrens Belo Horizonte MG Brasil Hospital Municipal Odilon Behrens,Belo Horizonte, MG – Brasil; 25 Hospital Moinhos de Vento Porto Alegre RS Brasil Hospital Moinhos de Vento,Porto Alegre, RS – Brasil; 26 Hospital Santa Cruz Santa Cruz do Sul RS Brasil Hospital Santa Cruz,Santa Cruz do Sul, RS – Brasil; 27 Hospital Regional Antônio Dias Patos de Minas MG Brasil Hospital Regional Antônio Dias,Patos de Minas, MG – Brasil; 28 Universidade Federal de Ouro Preto Ouro Preto MG Brasil Universidade Federal de Ouro Preto, Ouro Preto, MG – Brasil; 29 Hospital São Lucas PUCRS Porto Alegre RS Brasil Hospital São Lucas (PUCRS),Porto Alegre, RS – Brasil; 30 Hospital Márcio Cunha Ipatinga MG Brasil Hospital Márcio Cunha,Ipatinga, MG – Brasil; 31 Hospital Metropolitano Doutor Célio de Castro Belo Horizonte MG Brasil Hospital Metropolitano Doutor Célio de Castro,Belo Horizonte, MG – Brasil; 32 Hospital Semper Belo Horizonte MG Brasil Hospital Semper,Belo Horizonte, MG – Brasil

**Keywords:** COVID-19, Infecções por Coronavírus, Troponina, Respiração artificial, Mortalidade

## Abstract

**Fundamento:**

As complicações cardiovasculares da COVID-19 são aspectos importantes da patogênese e do prognóstico da doença. Evidências do papel prognóstico da troponina e da lesão miocárdica em pacientes hospitalizados com COVID-19 na América Latina são ainda escassos.

**Objetivos:**

Avaliar a lesão miocárdica como preditor independente de mortalidade hospitalar e suporte ventilatório mecânico em pacientes hospitalizados, do registro brasileiro de COVID-19.

**Métodos:**

Este estudo coorte é um subestudo do registro brasileiro de COVID-19, conduzido em 31 hospitais brasileiros de 17 cidades, de março a setembro de 2020. Os desfechos primários incluíram mortalidade hospitalar e suporte ventilatório mecânico invasivo. Os modelos para os desfechos primários foram estimados por regressão de Poisson com variância robusta, com significância estatística de p<0,05.

**Resultados:**

Dos 2925 pacientes [idade mediana de 60 anos (48-71), 57,1%], 27,3% apresentaram lesão miocárdica. A proporção de pacientes com comorbidades foi maior nos pacientes com lesão miocárdica [mediana 2 (1-2) vs. 1 (0-20)]. Os pacientes com lesão miocárdica apresentaram maiores valores medianos de peptídeo natriurético cerebral, lactato desidrogenase, creatina fosfoquinase, N-terminal do pró-peptídeo natriurético tipo B e proteína C reativa em comparação a pacientes sem lesão miocárdica. Como fatores independentes, proteína C reativa e contagem de plaquetas foram relacionados com o risco de morte, e neutrófilos e contagem de plaquetas foram relacionados ao risco de suporte ventilatório mecânico invasivo. Os pacientes com níveis elevados de troponina apresentaram um maior risco de morte (RR 2,03, IC95% 1,60-2,58) e suporte ventilatório mecânico (RR 1,87;IC95% 1,57-2,23), em comparação àqueles com níveis de troponina normais.

**Conclusão:**

Lesão cardíaca foi um preditor independente de mortalidade hospitalar e necessidade de suporte ventilatório mecânico em pacientes hospitalizados com COVID-19.

**Figura Central:**
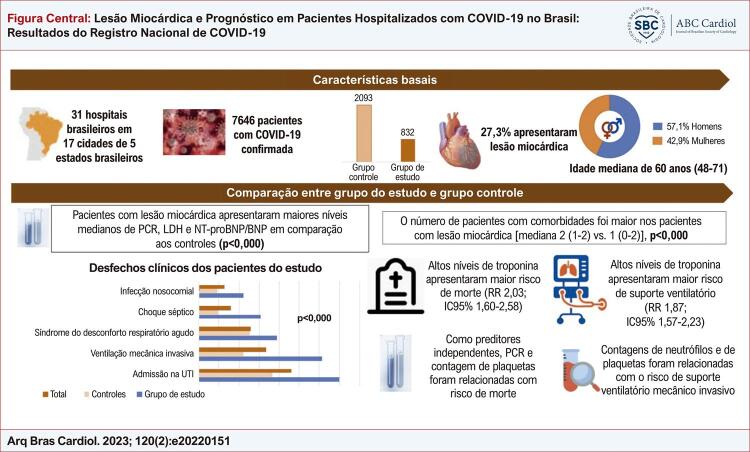
: Lesão Miocárdica e Prognóstico em Pacientes Hospitalizados com COVID-19 no Brasil: Resultados do Registro Nacional de COVID-19

Figura central dos principais resultados. BNP: peptídeo natriurético tipo B; LDH: lactato desidrogenase; NT-proBNP: N-terminal do pró-peptídeo natriurético tipo B; PCR: proteína C reativa; UTI: unidade de terapia intensiva.

## Introdução

Complicações cardiovasculares da doença causada pelo novo coronavírus (COVID-19)^1 , 2^ representam um aspecto importante da patogênese e do prognóstico da doença. A lesão miocárdica é comum em pacientes internados com COVID-19 e tem sido relatada em 7,2% a 36% de todos os pacientes.^3 - 6^ Embora essa condição tem sido associada a um pior prognóstico,^7^ evidências de seu papel prognóstico em pacientes internados com COVID-19 na América Latina ainda são escassas.

No Brasil, muitas pessoas ainda são afetadas pela doença cardíaca chagásica e valvulopatia reumática.^8^ Ainda, existem vários polimorfismos, heterogeneidade, e miscigenação na população, o que pode influenciar nas taxas de lesão miocárdica e níveis de biomarcadores em pacientes com COVID-19.^9^ Em uma análise prévia realizada pelo nosso grupo de pesquisa, nós desenvolvemos e validamos um escore com alta capacidade discriminatória para predizer mortalidade em pacientes brasileiros usando dados facilmente disponíveis na admissão dos pacientes, o escore ABC_2_-SPH.^10^

O presente estudo teve como objetivo avaliar a lesão miocárdica como um preditor independente de mortalidade hospitalar e suporte ventilatório mecânico invasivo em pacientes internados com COVID-19, a partir de dados do registro nacional.

## Materiais e métodos

Este estudo multicêntrico retrospectivo do tipo coorte é um subestudo do registro brasileiro de COVID-19, conduzido em 31 hospitais de 17 cidades de cinco estados (Minas Gerais, Pernambuco, Rio Grande do Sul, Santa Catarina e São Paulo), descrito em detalhes em um artigo anterior.^11^ O estudo foi aprovado pela Comissão Nacional de Ética em Pesquisa (CAAE: 30350820.5.1001.0008). Houve dispensa do consentimento livre e esclarecido individual devido à gravidade da situação imposta pela pandemia e à natureza retrospectiva do estudo.

Este estudo incluiu pacientes adultos consecutivos (idade ≥18 anos) com diagnóstico laboratorial confirmado de COVID-19,^12^ admitidos nos hospitais participantes entre março e setembro de 2020 e que possuíam pelo menos um valor de troponina. Foram excluídos pacientes com diagnóstico de insuficiência cardíaca crônica no prontuário médico, pacientes com taxa de filtração glomerular (TFG) menor que 30 mL/min/1,73m^2^ [estimada pela fórmula do *Chronic Kidney Disease Epidemiology Collaboration* (CKD-EPI), estabelecida por Levey et al.^13^ ), pacientes que receberam alta em menos de 24 horas, e aqueles diagnosticados com COVID-19 durante internação. Não calculamos tamanho amostral e todos os pacientes elegíveis foram incluídos.

### Coleta de dados

Dados demográficos, clínicos, de exames (laboratório, eletrocardiograma e ecocardiograma), de tratamentos, e desfechos foram coletados por professionais de saúde treinados e médicos residentes de cada centro, usando a ferramenta REDCap ( *Research Electronic Data Capture* versão 7.3.1),^14 - 16^ no Centro de Telessaúde do Hospital das Clínicas da Universidade Federal de Minas Gerais. Foram conduzidas verificações detalhadas para se assegurar a qualidade da coleta dos dados. Foi gerado um código no programa R para identificar inconformidades e inconsistências, conforme descrito anteriormente, com base em regras estabelecidas por experts, e cada centro foi contatado para verificar e corrigir os dados, se necessário.^10^

### Lesão miocárdica, teste da troponina e grupos de estudo

A lesão miocárdica foi definida como elevação da troponina cardíaca (cTnI ou cTnT) acima do limite superior de referência (URL) do percentil 99, de acordo com a quarta definição universal de universal de infarto do miocárdio.^17^ A medida da troponina foi realizada segundo decisão do médico responsável. Foram coletados os valores de troponina na admissão, bem como os valores mínimos e máximos após 24 horas de internação. Qualquer valor anormal entre essas medidas foi considerado para análise no presente estudo. O grupo de referência foi composto por pacientes com valores dentro da faixa de normalidade.

O teste de troponina variou entre os centros. Devido à falta de produtos químicos essenciais à realização do teste no início da pandemia, alguns centros usaram mais que um teste durante o período do estudo. Assim, na análise, considerou-se o URL do percentil 99 para cada teste para homens e mulheres, conforme descrito no Material Suplementar ( Tabela S1 ).

### Desfechos

Os desfechos primários foram mortalidade hospitalar e suporte de ventilação mecânica invasiva. Desfechos secundários incluíram complicações cardiovasculares (IC aguda, infarto do miocárdio agudo, e miocardite), sangramento, eventos tromboembólicos, choque séptico, coagulação intravascular disseminada, infecção nosocomial, admissão à unidade de terapia intensiva (UTI), tempo de internação da UTI, tempo de internação hospitalar, e necessidade de terapia renal substitutiva.

### Análise estatística

As análises estatísticas foram realizadas em três etapas principais: (i) descritiva, (ii) bivariada (avaliação da associação do desfecho com cada variável de interesse), e (iii) análises multivariadas.

Foram realizadas análises descritivas de todas as variáveis, estratificadas em grupo do estudo e grupo controle (pacientes sem infarto do miocárdio). As variáveis categóricas foram descritas como frequências absolutas e relativas. O teste de normalidade de Shapiro-Wilk foi conduzido para determinar se as variáveis contínuas apresentavam distribuição normal. Como todas as variáveis apresentaram distribuição não normal, elas foram descritas como medianas e intervalos interquartis (IIQ). O número de comorbidades foi definido baseado em oito comorbidades que se mostraram exercer um impacto prognóstico sobre a COVID-19 (hipertensão, diabetes mellitus, obesidade, doença arterial coronariana, *flutter* ou fibrilação atrial, cirrose, câncer e acidente vascular cerebral prévio).^10^

Na análise bivariada, variáveis clínicas e demográficas, e desfechos foram avaliados usando o teste exato de Fisher e o teste do qui-quadrado para comparar proporções, conforme apropriado. O teste de Kruskal-Wallis foi usado para comparar medianas das variáveis contínuas, e o teste de Dunn foi usado como um teste pós-hoc. Para as análises multivariadas, dois modelos preditivos foram estimados para avaliar o papel da troponina elevada sobre os desfechos primários: mortalidade hospitalar e suporte de ventilação mecânica invasiva. Todas as variáveis incluídas nos modelos foram obtidas na admissão hospitalar. Um conjunto de potenciais variáveis preditivas para os desfechos primários foi selecionado *a priori* (material suplementar – Figura S3 ) com base em evidências científicas de variáveis associadas com um pior prognóstico da COVID-19.^10^ Exames laboratoriais foram realizados à critério do médico assistente. Resultados de exames de imagens não foram incluídos, uma vez que esses exames nem sempre são realizados na admissão, e sua interpretação envolve o julgamento do examinador.

Os modelos para os desfechos primários foram estimados pela regressão de Poisson, com robusta variância. No modelo para predizer suporte ventilatório mecânico, os pacientes que estavam em ventilação mecânica invasiva na admissão não foram incluídos (n=72). A regressão de Poisson foi escolhida devido à sua capacidade de estimar o risco relativo (RR), que é o parâmetro de interesse primário, já que era esperada uma taxa elevada do evento.^18 , 19^

A modelagem dos modelos de predição dividiu as variáveis em cinco blocos pela abordagem *stepwise-forward*_,_^20^ mutualmente inseridas nos modelos de regressão um a cinco. Como o principal objetivo da análise foi identificar a associação da lesão miocárdica com os desfechos do estudo, a variável foi testada nos cinco modelos. O primeiro incluiu somente lesão miocárdica. O segundo adicionou idade e sexo, o terceiro adicionou o número de comorbidades, e o quarto adicionou características clínicas na admissão hospitalar. O quinto modelo multivariado continha somente as variáveis com significância estatística de 5% após ajuste quanto as outras variáveis adicionadas aos modelos multivariados anteriores. As variáveis foram incluídas da maior para a menor significância, para testar quais associações entre as variáveis explicativas e os desfechos continuariam significativos ao longo do processo.

A significância estatística das variáveis que eram parte dos modelos foi avaliada analisando-se o RR e seus respectivos intervalos de confiança de 95% (IC95%), bem como pelo valor p dos testes, objetivando-se reduzir a probabilidade do erro tipo I. Comparou-se a bondade do ajuste dos modelos usando Critério de Informação de Akaike.^20^

Para os modelos de regressão, foram estimados o RR e seus respectivos IC 95%. Todas as análises foram realizadas usando o programa STATA (StataCorp. 2012. Stata Statistical, versão 12) e o programa R (versão 4.0.2), usando os pacotes tidyverse, lubridate, stringi, rlang, jsonlite, Rcurl, writexl, openxlsx, readxl, e o pacote *sandwich* . Um valor de p <0,05 foi considerado estatisticamente significativo.

## Resultados

### Características basais

**Figura 1:**
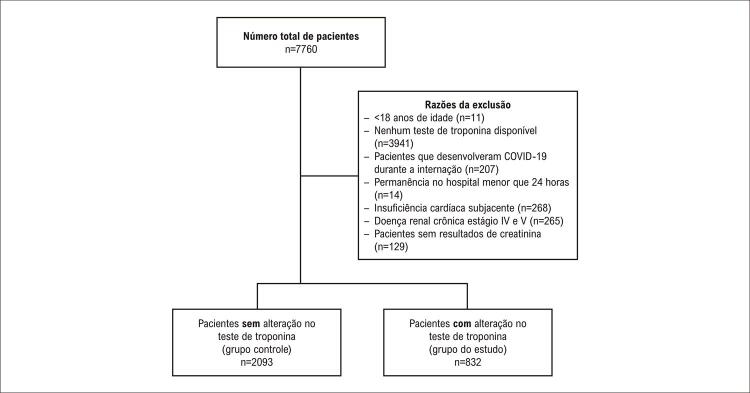
– Fluxograma dos pacientes incluídos no estudo.

Dos 7760 pacientes, 2925 foram incluídos nas análises presentes ( Figura 1 ). Características clínicas e demográficas são apresentadas na Tabela 1 . Pacientes com lesão miocárdica apresentaram idade mediana de 10 anos a mais que os controles, um maior número de comorbidades, e uma maior prevalência de doenças subjacentes – hipertensão, doença arterial coronariana, acidente vascular isquêmico, fibrilação atrial, diabetes, doença pulmonar obstrutiva crônica (DPOC), câncer e doença renal crônica (DRC). Ainda, houve uma maior proporção de indivíduos com lesão miocárdica com estado mental alterado e uma razão entre saturação periférica de oxigênio (SpO_2_) e a fração de oxigênio inspirado (FiO_2_) (razão SF) mais baixa na admissão. Em relação aos parâmetros laboratoriais, os pacientes com lesão miocárdica apresentaram maiores níveis medianos de proteína C reativa (PCR), lactado desidrogenase (LDH), N-terminal do pró-peptídeo natriurético tipo B(NT-proBNP)/ peptídeo natriurético tipo B (BNP) em comparação aos controles. Resultados do eletrocardiograma e do ecocardiograma estão descritos no material suplementar ( Tabela S2 ).

**Tabela 1:** – Características clínicas e demográficas da população do estudo na admissão

Variáveis	Grupo do estudo (n = 832) N (%)	Controles (n = 2093) N (%)	Total (n = 2925) N (%)	Valor p
Homens	456 (27,3%)	1213 (72,7%)	1669 (57,1%)	0,121
Idade *(mediana* )	67 (57-77)	57 (45-67)	60 (48-71)	0,000
**Comorbidades**				
Número total ( *mediana* )	2 (1-2)	1 (0-2)	1 (0-2)	0,000
**Doenças cardiovasculares**				
Hipertensão	533 (64,1%)	1,010 (48,3%)	1,543 (52,7%)	0,000
Doença arterial coronariana	62 (7,5%)	82 (3,9%)	144 (4,9%)	0,000
Acidente vascular cerebral isquêmico	42 (5,1%)	40 (1,9%)	82 (2,8%)	0,000
Fibrilação atrial / *flutter atrial*	38 (4,6%)	26 (1,2%)	64 (2,2%)	0,000
Doença de Chagas	2 (0,2%)	2 (0,1%)	4 (0,1%)	0,321
Doença valvar reumática	2 (0,2%)	0 (0,0%)	2 (0,1%)	0,081
**Doenças metabólicas**				
Diabetes mellitus	289 (34,7%)	528 (25,2%)	817 (27,9%)	0,000
Obesidade	167 (20,1%)	425 (20,3%)	592 (20,2%)	0,887
**Doenças respiratórias**				
Asma	149 (5,9%)	151 (7,2%)	200 (6,8%)	0,200
DPOC	75 (9,0%)	95 (4,5%)	170 (5,8%)	0,000
**Outras condições**				
Câncer	62 (7,5%)	78 (3,7%)	140 (4,8%)	0,000
Doença reumática	20 (2,4%)	32 (1,5%)	52 (1,8%)	0,106
Doença renal crônica	25 (3,0%)	14 (0,7%)	39 (1,3%)	0,000
HIV	5 (0,6%)	13 (0,6%)	18 (0,6%)	0,950
Cirrose	7 (0,8%)	4 (0,2%)	11 (0,4%)	0,016
**Características clínicas**	**(n = 832)**	**(n = 2,093)**	**(n = 2,925)**	
*Glasgow <15*	170 (20,4%)	156 (7,5%)	326 (11,1%)	0,000
	**(n = 810)**	**(n = 2,057)**	**(n = 2,867)**	
*Razão SF (mediana)*	362 (213-438)	433 (343-457)	424 (329-452)	0,000
**Pressão arterial sistólica**	**(n = 787)**	**(n = 1995)**	**(n = 2782)**	
≥90 (mmHg)	770 (97,8%)	1,997 (99,1%)	2,747 (98,7%)	0,003
<90 (mmHg)	9 (1,1%)	15 (0,7%)	24 (0,9%)	
Necessidade de inotrópicos	8 (1,0%)	3 (0,1%)	11 (0,4%)	
**Pressão arterial diastólica**	**(n = 796)**	**(n = 1989)**	**(n = 2785)**	
>60 (mmHg)	620 (77,9%)	1,737 (87,3%)	2,357 (84,6%)	0,000
≤60 (mmHg)	100 (12,6%)	210 (10,6%)	310 (11,1%)	
Necessidade de inotrópicos	76 (9,5%)	42 (2,1%)	118 (4,2%)	

*DPOC: doença pulmonar obstrutiva crônica; HIV: vírus da imunodeficiência humana; razãoSF: saturação periférica de oxigênio (SpO_*2*_ )/fração de oxigênio inspirado (FiO_*2*_ ).*

Os pacientes com lesão miocárdica apresentaram maior mortalidade hospitalar e maior frequência de desfechos secundários ( Tabela 2 ). Os modelos de regressão multivariada para mortalidade hospitalar e suporte de ventilação mecânica invasiva mostraram que a lesão miocárdica foi um importante preditor de ambos os desfechos, mesmo após ajuste para outras variáveis. Observou-se uma diminuição no impacto dos níveis elevados de troponina no modelo multivariado para mortalidade hospitalar ( Tabela 3 ) com a adição das variáveis ao modelo. No quinto modelo multivariado, os pacientes com níveis elevados de troponina apresentaram maior risco de morte em comparação aos controles (RR: 2,03 [1,60-2,58]). Idade, número de comorbidades, taxa respiratória na admissão, taxa SF e PCR da admissão também se associaram com um maior risco de morte. A lesão miocárdica também se mostrou um preditor independente de ventilação mecânica invasiva [RR: 1,87 (1,57-2,23)] ( Tabela 4 ). Houve associação do número de comorbidades, da taxa respiratória, PCR e do número de neutrófilos com um maior riso de suporte ventilatório mecânico. Por outro lado, razão SF elevada e contagem alta de plaquetas valores associaram-se com um menor risco de suporte ventilatório mecânico invasivo. Para informação sobre os modelos, ver material suplementar S4. A ilustração central representa a ilustração central dos principais resultados do artigo.

**Tabela 2:** – Desfechos clínicos dos pacientes do estudo

Variáveis	Grupo do estudo (n = 832) N (%)	Controles (n = 2093) N (%)	Total (n = 2925) N (%)	Valor p
**Avaliação clínica**				
Tempo de internação (dias) *(mediana)*	14 (7-25)	7 (4-13)	9 (5-16)	0,000
Admissão na UTI	584 (70,2%)	765 (36,6%)	1,349 (46,1%)	0,000
Tempo de internação na UTI (dias) *(mediana)*	12 (6-22)	8 (4-16)	10 (5-19)	0,000
Ventilação mecânica invasiva	509 (61,3%)	467 (22,3%)	976 (33,4%)	0,000
Síndrome do desconforto respiratório agudo	325 (39,1%)	428 (25,5%)	753 (25,7%)	0,000
Choque séptico	258 (31,0%)	201 (9,6%)	459 (15,7%)	0,000
Infecção nosocomial	186 (22,4%)	189 (9,0%)	375 (12,8%)	0,000
Hiperglicemia	148 (17,8%)	215 (10,3%)	363 (12,4%)	0,000
**Trombose vascular**	81 (9,7%)	125 (6,0%)	206 (7,1%)	0,000
Trombo- embolismo pulmonar	57 (6,9%)	95 (4,5%)	152 (5,2%)	0,011
Trombose venosa profunda	25 (3,0%)	31 (1,5%)	56 (1,9%)	0,007
Trombose arterial	6 (0,7%)	4 (0,2%)	10 (0,3%)	0,027
Insuficiência cardíaca aguda ou descompensada	33 (4,0%)	23 (1,1%)	56 (1,9%)	0,000
Infarto agudo do miocárdio	25 (3,0%)	2 (0,1%)	27 (0,9%)	0,000
Miocardite	7 (0,8%)	2 (0,1%)	9 (0,3%)	0,001
Sangramento	26 (53,1%)	24 (46,9%)	49 (1,7%)	0,000
Coagulação intravascular disseminada	7 (0,8%)	6 (0,3%)	13 (0,4%)	0,042
Morte	376 (45,2%)	218 (10,4%)	594 (20,3%)	0,000

*UTI: unidade de terapia intensiva.*

**Tabela 3:** – Preditores (na admissão hospitalar) de mortalidade hospitalar pelo modelo de regressão de Poisson com variância robusta

Variáveis	Modelo multivariado 1 RR (IC95%)	Modelo multivariado 2 RR (IC95%)	Modelo multivariado 3 RR (IC95%)	Modelo multivariado 4 RR (IC95%)	Modelo multivariado 5 RR (IC95%)
Lesão miocárdica	4,2482 (3,2820-5,4982)*	3,1956 (2,4335- 4,1962)*	3,1077 (2,4364- 3,9640)*	1,9057 (1,4843-2,4468)*	2,0323 (1,5995-2,5822)*
Idade	---	1,0285 (1,0191-1,0379)*	1,0273 (1,0190- 1,0357)*	1,0262 (1,0176-1,0349)*	1,0269 (1,0192-1,0348)*
Sexo feminino	---	0,8122 (0,6289-1,0489)***	0,7924 (0,6461-0,9718)**	0,9553 (0,7736-1,2621)***	---
Número de comorbidades ****	---	---	1,1550 (1,0317- 1,2930)**	1,1089 (1,0187-1,2070)*	8,1862 (7,5153-8,9168)*
Taxa respiratória	---	---	---	1,0213 (1,0018-1,0429)**	1,0223 (1,0086-1,0362)**
Frequência cardíaca	---	---	---	1,0039 (0,9994-1,0083)***	---
Pressão arterial sistólica <90 mmHg sem inotrópico	---	---	---	0,2508 (0,1062-0,5920)**	---
Pressão arterial sistólica <90 mmHg com inotrópico	---	---	---	1,3022 (0,7688-2,2056)***	---
Glasgow <15	---	---	---	1,0723 (0,7372-1,5599)***	---
Razão SF	---	---	---	0,9971 (0,9959-0,9983)*	0,9969 (0,9963-0,9977)*
Ventilação mecânica invasiva	---	---	---	0,9714 (0,5843-1,6148)***	---
Proteína C reativa	---	---	---	1,0010 (1,0006-1,0027)*	1,0020 (1,0009-1,0026)*
Hemoglobina	---	---	---	1,0150 (0,9644-1,0682)***	---
Neutrófilos	---	---	---	1,0000 (1,0000-1,0000)*	---
Contagem de plaquetas	---	---	---	0,9994 (0,9993-0,9996)*	0,9994 (0,9993-0,9999)*
Ureia	---	---	---	1,0046 (1,0001-1,0103)**	---
Lactato	---	---	---	0,9775 (0,9421-1,0143)***	---
Sódio	---	---	---	0,9948 (0,9763-1,0136)***	---
Bicarbonato	---	---	---	0,9716 (0,9443-0,9996)*	---
pH	---	---	---	0,9987 (0,9982-0,9993)*	---
pCO_2_	---	---	---	1,0034 (0,9939-1,0129)***	---
D-dímero	---	---	---	0,9999 (0,9998-1,0001)***	---

IC: intervalo de confiança; RR: risco relativo; razão SF: razão entre saturação periférica de oxigênio (SpO_2_)/fração de oxigênio inspirado (FiO_2_); *p<0,0001; **p<0,05; ***p>0,05; ****Hipertensão, diabetes mellitus, obesidade, doença arterial coronariana, insuficiência cardíaca, fibrilação ou flutter atrial, cirrose, câncer e acidente vascular cerebral prévio.

**Tabela 4:** – Preditores (na admissão hospitalar) de mortalidade hospitalar pelo modelo de regressão de Poisson com variância robusta

Variáveis	Modelo multivariado 1 RR (IC95%)	Modelo multivariado 2 RR (IC95%)	Modelo multivariado 3 RR (IC95%)	Modelo multivariado 4 RR (IC95%)	Modelo multivariado 5 RR (IC95%)
Lesão miocárdica	2,8607 (2,4221-3,3787)*	2,6906 (2,1353- 3,3901)*	2,5901 (2,1627-3,1021)*	1,9018 (1,5842-2,2830)*	1,8675 (1,5662-2,2268)*
Idade	---	1,0057 (0,9983-1,0131)***	1,0034 (0,9976-1,0093)***	1,0068 (1,0002-1,0133)*	---
Sexo feminino	---	0,8889 (0,7492-1,0546)***	0,8697 (0,7492-1,0546)***	0,9398 (0,7852-1,1249)***	---
Comorbidades ****	---	---	1,1781 (1,0936-1,2691)*	1,1313 (1,0494-1,2196)**	1,1295 (1,0488-1,2164)*
Taxa respiratória	---	---	---	1,0369 (1,0254-1,0485)*	1,0332 (1,0222-1,0443)*
Frequência cardíaca	---	---	---	1,0002 (0,9954-1,0051)***	---
Pressão arterial sistólica < 90 mmHg sem inotrópicos	---	---	---	0,4001 (0,1252-1,2781)***	---
Glasgow <15	---	---	---	0,8320 (0,6109-1,1332)***	---
Razão SF	---	---	---	0,9980 (0,9960-0,9990)*	0,9980 (0,9970-0,9990)*
Proteína C-reativa	---	---	---	1,0027 (1,0019-1,0034)*	1,0030 (1,0020-1,0040)*
Hemoglobina	---	---	---	1,0111 (0,9641-1,0605)***	---
Neutrófilos	---	---	---	1,0005 (1,0003-1,0006)*	1,0004 (1,0002-1,0005)*
Contagem de plaquetas	---	---	---	0,9980 (0,9970-0,9990)*	0,9995 (0,9994-0,9997)*
Ureia	---	---	---	0,9954 (0,9912-0,9997)**	---
Lactato	---	---	---	0,9740 (0,9045-1,0487)***	---
Sódio	---	---	---	0,9869 (0,9688-1,0054)***	---
Bicarbonato	---	---	---	1,0439 (0,9752-1,1174)***	---
pH	---	---	---	0,0965 (0,0039-2,3727)***	---
pCO_2_	---	---	---	0,9743 (0,9413-1,0084)***	---
D-dímero	---	---	---	0,9999 (0,9998-1,0000)***	---

IC: intervalo de confiança; RR: risco relativo; razão SF: razão entre saturação periférica de oxigênio (SpO_*2*_ )/fração de oxigênio inspirado; *p<0,0001; **p<0,05; ***p>0,05; ****Hipertensão, diabetes mellitus, obesidade, doença arterial coronariana, insuficiência cardíaca, fibrilação ou flutter atrial, cirrose, câncer e acidente vascular cerebral prévio.

## Discussão

Neste estudo coorte multicêntrico, incluindo uma grande amostra de 31 hospitais brasileiros, os pacientes com lesão miocárdica eram 10 anos (mediana) mais velhos que os indivíduos controles, apresentaram uma maior prevalência de comorbidades, piores parâmetros clínicos na admissão hospitalar, e níveis mais altos de PCR, LDH e NT-proBNP/BNP em comparação àqueles sem lesão miocárdica. A lesão miocárdica foi um preditor independente de mortalidade hospitalar [RR: 2,03 (1,60-2,58)] e suporte ventilatório mecânico invasivo [RR: 1,87 (1,57-2,23)]. Um maior número de comorbidades, maior taxa respiratória, e maiores níveis de PCR foram associados com um maior risco de mortalidade hospitalar e suporte ventilatório mecânico invasivo. Ainda, idade e uma razão SF mais baixa mostraram associação independente com mortalidade hospitalar, enquanto o número de neutrófilos associou-se com um risco aumentado de suporte ventilatório mecânico. Os pacientes com lesão miocárdica mostraram uma maior frequência de complicações cardiovasculares, sangramentos, eventos tromboembólicos, choque séptico, coagulação intravascular disseminada, infecção nosocomial, admissão à UTI, tempo de internação hospitalar e na UTI, e necessidade de terapia renal substitutiva.

Os achados do presente estudo estão de acordo com os resultados de estudos de outros países, descrevendo características muito similares de pacientes com COVID-19 que desenvolveram lesão miocárdica, incluindo idade avançada e uma alta prevalência de condições médicas subjacentes.^6 , 21 - 23^ Não houve evidência de diferenças de sexos na prevalência das variáveis analisadas nos pacientes com lesão miocárdica, o que contrasta com dados publicados anteriormente na literatura. Na revisão sistemática realizada por Toraih et al.,^24^ incluindo 17794 pacientes com lesão cardíaca avaliada por medida de troponina, os autores encontraram proporção significativamente maior de homens com doença crítica, definida como síndrome do desconforto respiratório agudo, ventilação mecânica invasiva, e admissão na UTI. Na presente análise, mulheres mais velhas com lesão miocárdica mostraram um risco significativamente maior de morte e maior necessidade (não estatisticamente significativa) de ventilação mecânica invasiva.

De fato, os níveis de troponina e de outros biomarcadores são geralmente anormais em pacientes internados com COVID-19. Há diferentes mecanismos para o desenvolvimento de lesão cardíaca em pacientes com COVID-19, tais como esforço cardíaco aumentado na insuficiência respiratória aguda,^25^ e interação do SARS-CoV-2 com receptores da enzima conversora de angiotensina 2.^2 , 26 - 28^ Embora os mecanismos específicos da lesão miocárdica sejam incertos, os mecanismos propostos incluem resposta inflamatória e distúrbios do sistema imune durante a progressão da doença.^26 , 29^

Resultados dos testes usados para avaliar função e lesão cardíaca mostraram valores significativamente maior nos pacientes com COVID-19 que morreram que nos pacientes que receberam alta com vida. Nossos resultados corroboram esses achados, uma vez que BNP, creatina fosfoquinase, LDH, NT-proBNP, e PCR, um biomarcador da resposta inflamatória, eram mais altos nos pacientes com lesão miocárdica. Um estudo recente que incluiu 187 pacientes com COVID-19 hospitalizados no Rio de Janeiro também mostrou que a troponina foi um preditor independente de eventos adversos. Já o BNP não foi um preditor independente de mortalidade ou necessidade de suporte ventilatório mecânico.^29^ Esse achado pode ser limitado pelo pequeno tamanho amostral e alto número de valores faltantes.

Uma revisão sistemática e meta-análise de Alzahrani e Al-Rabia,^2^ de estudos chineses do tipo coorte, mostrou que 45,2% dos pacientes com lesão miocárdica induzida por COVID-19 morreram.^26^ No presente estudo, a taxa de mortalidade foi 4,25 vezes maior nos pacientes com lesão miocárdica em comparação aos controles, além de um tempo mediano mais longo de internação hospitalar, e maior ocorrência de admissão na UTI e dos outros desfechos secundários mencionados anteriormente. Assim, os pacientes com COVID-19 e lesão cardíaca eram mais susceptíveis a complicações da doença e apresentaram piores diagnósticos. Esses achados são consistentes com os resultados descritos em estudos conduzidos em outros países.^6 , 31 , 32^

Atualmente, vários estudos propuseram variáveis clínicas, achados laboratoriais e de radiografia do tórax para a predição do risco de progressão da COVID-19 grave e mortalidade.^33^ Em uma análise prévia, nosso grupo de pesquisa desenvolveu e validou um escore de predição de risco para mortalidade hospitalar em pacientes com COVID-19 (disponível em https://abc2sph.com/pt/), que inclui idade mais avançada, nitrogênio ureico no sangue, número de comorbidades, PCR, razão SF periférica, contagem de plaquetas, e frequência cardíaca como preditores. No entanto, a troponina não foi incluída em nossas análises.^10^ No presente estudo, a lesão cardíaca foi um fator de risco independente para mortalidade e suporte de ventilação mecânica invasiva, além das variáveis testadas anteriormente, que foi o principal achado das análises presentes. Esses resultados exercem um importante papel no cuidado do paciente, uma vez que podem auxiliar os profissionais da saúde a identificarem pacientes que possam ter um pior prognóstico, direcionando intervenções para o manejo das condições clínicas e melhorias no cuidado da saúde. Uma próxima etapa seria avaliar a inclusão da troponina ao escore de risco ABC_2_-SPH, um importante tópico para estudos futuros sobre ABC_2_-SPH. Esperamos que esta coorte também possa ser útil para estudos futuros no desenvolvimento de um escore preditivo para lesão miocárdica.

Este estudo possui limitações. Primeiramente, não podemos assegurar representatividade nacional dos hospitais. Uma vez que este estudo se trata de uma análise multicêntrica da COVID-19, os testes de troponina poderiam apresentar inconsistências, uma vez que múltiplos kits laboratoriais comerciais foram usados de modo interinstitucional e intrainstitucional, e diferentes valores de referência foram padronizados em cada estudo, levando ao viés de medida. Outra limitação foram as análises agrupadas que não permitiram o reconhecimento da contribuição de cada centro aos desfechos de morte e suporte ventilatório mecânico, bem como fatores institucionais relacionados à mortalidade.^34^ É importante mencionar que o período da pandemia quando os dados foram coletados envolveu uma população que não havia sido vacinada,^35^ e parte dos pacientes do grupo de estudo podem não ter desenvolvido lesão miocárdica, e sim infarto agudo do miocárdio. Devido ao risco potencial de transmissão da COVID-19, existe a possibilidade de que nenhuma técnica diferencial diagnóstica seja realizada em alguns casos.^36^ Ainda, uma vez que o presente estudo foi um estudo observacional, outras variáveis potencialmente não mensuráveis e de confusão ou não reconhecidas podem não ter sido coletadas ou analisadas.

Quanto aos pontos fortes do estudo, um critério metodológico rigoroso foi usado neste estudo, o qual se baseou em uma amostra robusta de paciente, com diagnóstico confirmado de COVID-19. A amostra foi obtida com a colaboração de pesquisadores de 31 hospitais públicos, privados ou mistos, de diferentes tamanhos e níveis de complexidade, de diferentes regiões para garantir a diversidade da população estudada. Contudo, esses hospitais não são representativos de todo o sistema de saúde do país.

## Conclusão

A lesão cardíaca, medida pelos níveis de troponina elevados, foi um preditor independente de mortalidade e suporte ventilatório mecânico em pacientes internados com COVID-19, assim como as variáveis de um escore de predição de risco recentemente validado. Estratégias futuras envolvimento um monitoramento frequente dos níveis de troponina como biomarcadores de risco em pacientes com COVID-19 durante a hospitalização devem ser testadas para investigar seu papel na redução de risco de complicações e morte, e na melhoria do cuidado do paciente.

## *Material suplementar

Para informação adicional, por favor,clique aqui


