# Supplementary material for: Lesão Miocárdica e Prognóstico em Pacientes Hospitalizados com COVID-19 no Brasil: Resultados do Registro Nacional de COVID-19
Source: Arq Bras Cardiol. 2023 Feb 13;120(2):e20220151. [Article in Portuguese] doi: 10.36660/abc.20220151 (PMC10263463; doi:10.36660/abc.20220151)
Supplement: Supplementary file 1 [file 2022-0151_AO_R1_Supplementarymaterial.pdf]

MYOCARDIAL INJURY AND PROGNOSIS IN HOSPITALIZED BRAZILIAN COVID-19 PATIENTS: RESULTS FROM THE BRAZILIAN COVID-19 MULTI-CENTRICAL REGISTER

Myocardial injury and prognosis in COVID-19 patient

Supplementary material

Table S1 - Description of the methods employed to measure troponin levels by each study setting

Code center	Method	Equipment	Reference values	Measurement unit	Comments
1001	Homogeneous chemiluminescent immunoassay	Siemens Dimension EXL	Male: < 74,9<br>Female: < 51,1	pg/mL	High-Sensitivity Troponin I
1002	Homogeneous chemiluminescent immunoassay	Siemens Dimension EXL	Male: < 74,9<br>Female: < 51,1	pg/mL	High-Sensitivity Troponin I
1003	Chemiluminescence	Vitros Ortho clinical	Male: < 12,0 Female: < 9,0	ng/L	Ultra-Sensitive Troponin I
1005	Fluorescent immunoassay	Celer Finecare FIA Meter	≤ 0,30	ng/mL	Troponin I conventional
1006	Amplified Chemiluminescence	VITROS 3600	< 0,120	ng/mL	High-Sensitivity Troponin I

	Amplified Chemiluminescence	Vitros ECI	≤ 11,0	ng/L	High-Sensitivity Troponin I
	Chromatographic immunoassay	Medteste MedLevensohn	Reactive and non-reactive	Qualitative	Qualitative detection of Troponin I
1007	Electrochemiluminescence immunoassay	Cobas E411 Roche	< 0,30	ng/mL	Troponin I conventional
	Electrochemical enzyme-linked immunosorbent Assay	i-STAT<br>cardiac troponin I	< 0,08	ng/mL	Troponin I conventional
1008	Chemiluminescence microparticle immunoassay	Architect i-1000 (Abbott)	< 34,2	ng/mL	High-Sensitivity Troponin I
	Direct chemiluminometric	Atellica IM High Sensitivity Troponin I	≥ 28,0 ≤ 78,0	ng/L	Ultra-Sensitive Troponin I
	Homogeneous Immunochemiluminescence	Siemens Dimension EXL	Male: < 74,9<br>Female: < 51,1	pg/mL	High-Sensitivity Troponin I
1009	Chemiluminescence microparticle immunoassay	Architect ci 4100	Male: < 34,2<br>Female: < 15,6	pg/mL	High-Sensitivity Troponin I
1010	Chemiluminescence microparticle immunoassay	Architect i2000SR	Male: < 0,034<br>Female: < 0,015	ng/mL	Ultra-Sensitive Troponin I
1011	Enzyme-linked fluorescent assay	Vidas Biomerieux	Male: < 25<br>Female: < 11	ng/L	High-Sensitivity Troponin I

1012	Fluorescent immunoassay	Ichroma II	< 0,30	ng/mL	Troponin I conventional
1013	Chemiluminescence	Beckmann Coulter Access 2<br>Vitros	< 0,30	pg/mL	Ultra-Sensitive Troponin I
1014	Electrochemiluminescence	Coba h 232	< 100,00	ng/mL	Troponin T conventional
1015	Fluorescent immunoassay	Celer Finecare FIA Meter	< 0,30	ng/mL	Troponin I conventional
1016	Direct chemiluminescence	Advia Centaur XP - Siemens.	< 47,0	ng/L	Ultra-Sensitive Troponin I
	Enzyme-linked fluorescent assay	Vidas Biomerieux	< 19,0	ng/L	High-Sensitivity Troponin I
1017	Enzyme-linked fluorescent assay	Mini Vidas STAT	< 2,0	ng/L	High-Sensitivity Troponin I
1018	Electrochemiluminescence immunoassay	Cobas E411 Roche	≤ 0,30	ng/mL	Troponin I conventional
1019	Electrochemiluminescence	Elecsys	< 14,0	ng/L	Ultra-Sensitive Troponin T
1020	Chemiluminescence	Access 2	Male: < 19,8 ou<br>Female: < 11,6 ou	pg/mL	High-Sensitivity Troponin I

1021	Electrochemiluminescence	Elecsys	< 14,0	ng/L	Ultra-Sensitive Troponin T
	Amplified Chemiluminescence	VITROS 3600	< 0,120	ng/mL	High-Sensitivity Troponin I
1022	Amplified Chemiluminescence	Vitros ECI	≤ 11,0	ng/L	High-Sensitivity Troponin I
	Chromatographic immunoassay	MedLevensohn Medteste	Reactive and non-reactive	Reactive and non-reactive	Qualitative detection of Troponin I
1024	Electrochemiluminescence	Elecsys	< 14,0	ng/L	Ultra-Sensitive Troponin T
1025	Amplified Chemiluminescence	VITROS 3600	< 0,120	ng/mL	High-Sensitivity Troponin I
	Amplified Chemiluminescence	Vitros ECI	≤ 11,0	ng/L	High-Sensitivity Troponin I
1026	Chemiluminescence	Architect i2000	Male: < 0,1392 Female: < 0,01175	ng/ml	Troponin I conventional
1027	Electrochemiluminescence	Elecsys	< 0,014	ng/ml	Ultra-Sensitive Troponin T
1028	Chemiluminescence	Architec plus I1000SR	Male: < 34,2<br>Female: < 15,6	pg/mL	High-Sensitivity Troponin I

1029	Chemiluminescence	Vitros ECI	≤ 11,0	ng/L	Ultra-Sensitive Troponin I
1030	Chemiluminescence microparticle immunoassay	Architect Stat	≥ 0 ≤ 27	pg/mL	High-Sensitivity Troponin I
1031	Chromatographic immunoassay	ECO Teste - TR.3002C	> 1,0	ng/mL	Troponin I conventional
1032	Chemiluminescence microparticle immunoassay	Architect stat	≥ 0 ≤ 27	pg/mL	High-Sensitivity Troponin I
1035	Chemiluminescence microparticle immunoassay	Architect stat	≥ 0 ≤ 27	pg/mL	High-Sensitivity Troponin I
1036	Electrochemiluminescence	Elecsys	< 14	pg/mL	High-Sensitivity Troponin T
1037	Chemiluminescent with Acridinium Ester	Advia Centaur	< 0,04	ng/mL	High-Sensitivity Troponin I
1038	Chemiluminescence	Architect I 2000	Male: ≤ 34,2 and Female: ≤ 15,6	pg/mL	Ultra-Sensitive Troponin I

Table S2. Complementary cardiac and vascular evaluations of study patients

	STUDY GROUP N (%)	CONTROL PATIENTS N (%)	TOTAL N (%)	*p-value*
**ECG in the first 24 hours of admission?**	**(n= 832)**	**(n= 2.093)**	**(n= 2.925)**	
1. Yes	310 (37.3%)	636 (30.4%)	946 (32.3%)	0.0000
0. No	522 (62.7%)	1.457 (69.6%)	1.979 (67.7%)	
	**(n= 94)**	**(n= 244)**	**(n= 338)**	
**ECG QTc interval (ms)** *(median)*	0.44 (0.39-413)	0.42 (0.4-395)	0.42 (0.4-399)	0.5039*
	**(n= 69)**	**(n= 181)**	**(n= 250)**	
**ECG QT interval (ms)** *(median)*	360 (330-400)	360 (320-391)	360 (320-400)	0.0889*
**ECG Rhythm**	**(n= 309)**	**(n= 633)**	**(n= 942)**	
1. Sinusal	245 (79.3%)	576 (91.0%)	821 (87.1%)	0.0000
2. Atrial fibrillation or *flutter*	25 (8.1%)	20 (3.2%)	45 (4.8%)	0.0010
3. Artificial pacemaker	4 (1.3%)	1 (0.2%)	5 (0.5)	0.0420*
4. Supraventricular tachycardia	4 (1.3%)	2 (0.3%)	6 (0.6%)	0.0940*
5. Monomorphic ventricular tachycardia	0 (0.0%)	1 (0.2%)	1 (0.1%)	1.0000*
6. Others	32 (10.4%)	39 (6.2%)	71 (7.5%)	0.0220
**ECG changes**	**(n= 309)**	**(n= 631)**	**(n= 940)**	
1. Primary repolarization changes	71 (23.0%)	169 (26.8%)	240 (25.5%)	0.2090*
2. Right bundle branch block	12 (3.9%)	28 (4.4%)	40 (4.3%)	0.693*
3. Left bundle branch block	16 (5.2%)	15 (2.4%)	31 (3.3%)	0.0240
4. First degree AV block	5 (1.6%)	16 (2.5%)	21 (2.2%)	0.3710*
5. Complete AV block	3 (1.0%)	0 (0.0%)	3 (0.3%)	0.0350
6. Left anterior hemiblock	15 (4.9%)	13 (2.1%)	28 (3.0%)	0.0180
7. Pathologic Q waves	4 (1.3%)	4 (0.6%)	8 (0.9%)	0.4500*
8. Left ventricular overload with ST-T abnormalities	13 (4.2%)	7 (1.1%)	20 (2.1%)	0.0020
9. None of the above	198 (64.1%)	404 (64.0%)	602 (64.0%)	0.987*
	**(n= 246)**	**(n= 545)**	**(n= 791)**	
**Heart Rate (bpm)** *(median)*	88 (75-102)	84 (74-96)	86 (74-98)	0.0177*
**Echocardiogram in the first 24 hours of admission?**	**(n= 832)**	**(n= 2.092)**	**(n= 2.924)**	
1. Yes	65 (7.8%)	57 (2.7%)	122 (4.2%)	0.0000
0. No	767 (92.2%)	2.035 (97.3%)	2.802 (95.8%)	
	**(n= 62)**	**(n= 52)**	**(n= 114)**	
**Ejection fraction in the first 24 hours (%)** *(median)*	64 (58-68)	64 (61-68)	64 (60-68)	0.5883*
**Segmental alteration in the first 24 hours of admission?**	**(n= 65)**	**(n= 56)**	**(n= 121)**	
1. Yes	9 (13.9%)	4 (7.1%)	13 (10.7%)	0.3780*
0. No	56 (86.1%)	52 (92.9%)	108 (89.3%)	
**Type of segmental alteration in the first 24 hours of admission?**	**(n= 832)**	**(n= 2.093)**	**(n= 2.925)**	
1. Akinesia	2 (0.2%)	0 (0.0%)	2 (0.1%)	0.0810*
2. Dyskinesia	0 (0.0%)	1 (0.1%)	1 (0.0%)	1.000*
3. Bradykinesia	7 (0.8%)	3 (0.1%)	10 (0.3%)	0.0080

**Echocardiogram performed after 24 hours of admission?**	**(n= 832)**	**(n= 2.088)**	**(n= 2.920)**	
1. Yes	201 (24.2%)	236 (11.3%)	437 (15.0%)	0.0000
0. No	631 (75.8%)	1.852 (88.7%)	2.483 (85.0%)	
	**(n= 178)**	**(n= 219)**	**(n= 397)**	
**Ejection fraction after 24 hours from admission? (%)** *(median)*	64 (58-69)	65 (61-68)	65 (60-68)	0.1107*
**Segmental change after 24 hours of admission?**	**(n= 201)**	**(n= 236)**	**(n= 437)**	
1. Yes	20 (9.9%)	9 (3.8%)	29 (6.6%)	0.0100
0. No	181 (90.1%)	227 (96.2%)	408 (93.4%)	
**Type of segmental alteration after 24 hours from admission?**	**(n= 832)**	**(n= 2.093)**	**(n= 2.925)**	
1. Akinesia	8 (1.0%)	3 (0.1%)	11 (0.4)	0.0030
2. Dyskinesia	1 (0.1%)	4 (0.2%)	5 (0.2%)	1.0000*
3. Bradykinesia	11 (1.3%)	2 (0.1%)	13 (0.4%)	0.0000

*p>0.050
Abbreviations: ECG: electrocardiogram; AV: atrioventricular; bmp: beats per minute.
Numbers are expressed as n(%) or median (IQR).

Figure S3. Figure of the candidate predictor variables for the outcomes (death and invasive mechanical ventilation support)

Variables	Code	Blocks			
troponin	estudo	1			
Sex at birth	sexo				
Age (years)	idade		2		
Number of comorbidities	n_comorbidades_2			3	
Respiratory rate (irpm)	fr_adm				
Heart rate (bpm)	fc_adm				
Systolic blood pressure (mm Hg)	pasamina90				
Glasgow coma score	glasgow_menor15				
SF ratio	sat_fio2				
Mechanical ventilation	vm_adm				
C reactive protein (mg/L)	pcr_adm				
Hemoglobin (g/L)	hb_adm				
Neutrophils-to-lymphocytes ratio	nentrofilos_adm				
Platelet count (109/L)	plaquetas_adm				
Urea (mg/dL)	ureia_adm				
Lactate (mmol/L)	lactato_adm_valor				
Sodium (mmol/L)	sodio_adm				
Bicarbonate (mEq/L)	bicarbonato_adm				
pH	ph_adm				
pCO_2 (mmHg)	pco2_adm				
D-dimer	dimero_adm				4

Supplementary material S4. Detailed description of methods employed to test multivariate models by mortality and mechanical ventilation

Mortality model: There was a decrease in the effect of troponin on mortality as variables were added to the model. In model 1, in which we evaluated the isolated effect of troponin on mortality, we found a 4.25 higher risk of mortality in patients with altered troponin compared to the control group. By adding the variables age and sex at birth, there was a reduction in the relative risk of troponin in relation to the outcome of interest (RR=3.20), there was a 3% increase in the risk of mortality with each year of increase in age, conversely there was a reduction in the risk for women relative to men (RR=0.81). In model 3, when adding the number of comorbidities, we observed little reduction in the effect of troponin compared to model 2. In this model, the risk of mortality for patients with altered troponin was 3.11, with increased risk with advancing age (RR=1.03) and the number of comorbidities (RR=1.16), and with reduced risk for women compared to men (RR=0.79). However, in model 4 there was a significant reduction in the effect of troponin on the outcome (RR=1.91) when clinical variables were included together with the sociodemographic variables and number of comorbidities. In this model many variables were not statistically significant: sex, heart rate on admission, Glasgow index, mechanical ventilation on admission, hemoglobin on admission, lactate on admission, hemoglobin upon admission, pCO_2 upon admission and D-dimer on admission (Table 3). Model 5 contains only the variables that remained significant at the 5% level after adjustment for the other variables. In this model, patients with altered troponin have a higher risk of mortality (RR=2.030) compared to patients without alterations in this marker, age (RR=1.03), number of comorbidities (RR=1.100), respiratory rate on admission (RR=1.020), SF ratio (RR=0.996), C-reactive protein upon admission (RR=1.002) remained as predictors of the occurrence of mortality (Table 3).

Mechanical ventilation model: The association of troponin with the outcome of mechanical ventilation showed little reduction when comparing models 1 (RR=2.86), models 2 (RR=2.69) and model 3 (RR=2.69). In model 2 there was an increase in the risk of ventilation for patients with altered troponin and 0.10% for each year of increase in the age of patients (RR=1.010), inversely for females there was a reduction in risk in relation to men (RR=0.89). In model 3 when we inserted the variable number of comorbidities, the variables altered troponin and number of comorbidities (RR=1.180) remained associated with the need for mechanical ventilation (Table 4). In adjusting model 4 with the inclusion of clinical variables together with the variables in model 3, we observed a reduction in the effect of troponin on the outcome (RR=1. 900), in this model many variables were not statistically significant at the 5% level: sex, pasamine90, glasgow_minor15, hb on admission, lactate_adm_value, sodium_adm, bicarbonate_adm, ph_adm, pco2_adm and dimero_adm (Table 4). In model 5, we observed increased risk of mechanical ventilation in patients with altered troponin (RR=1.870), there was 13% increase in risk with increased number of comorbidities (RR=1.130), 0.30% with increased respiratory rate on admission (RR=1.030), 0.03% with increased C-reactive protein upon admission (RR=1.003) and 0.004% with increased number of neutrophils (RR=1.0004) and reduced risk for SF ratio (RR=0.995) and platelet count (RR=0.993) (Table 4).

Supplementary material S5. Letter attesting to proofreading and services

July 25, 2022

To Whom It May Concern,

I do hereby attest to the fact that the article, "**MYOCARDIAL INJURY AND PROGNOSIS IN HOSPITALIZED BRAZILIAN COVID-19 PATIENTS: RESULTS FROM THE BRAZILIAN COVID-19 REGISTRY**", has been proofread and revised by me, and that no grammatical or vocabulary errors exist within this text. Any further claims regarding grammatical and/or vocabulary errors should be specifically pinpointed and highlighted within the text with a suggested change by the reviewer for due contemplation of its validity and accuracy.

Sincerely,

Todd Irwin Marshall
Ph.D. – University of North Carolina
ELT Consultant and Executive Director
Pennsylvania English Consultoria e Traduções LTDA
(5531) 3423-1690
(5531) 9959-8751
todd@toddmarshall.com.br
www.toddmarshall.com.br
