# Supplementary material for: Lesão Miocárdica e Prognóstico em Pacientes Hospitalizados com COVID-19 no Brasil: Resultados do Registro Nacional de COVID-19
Source: Arq Bras Cardiol. 2023 Feb 13;120(2):e20220151. [Article in Portuguese] doi: 10.36660/abc.20220151 (PMC10263463; doi:10.36660/abc.20220151)
Supplement: Translation

# Translation: Myocardial Injury and Prognosis in Hospitalized COVID-19 Patients in Brazil: Results From The Brazilian COVID-19 Registry

**Authors:** Hannah Cardoso Barbosa, Maria Auxiliadora Parreiras Martins, Jordana Cristina de Jesus, Karina Cardoso Meira, Luiz Guilherme Passaglia, Manuela Furtado Sacioto, Adriana Falangola Benjamin Bezerra, Alexandre Vargas Schwarzbold, Amanda de Oliveira Maurílio, Barbara Lopes Farace, Carla Thais Cândida Alves da Silva, Christiane Corrêa Rodrigues Cimini, Daniel Vitorio Silveira, Daniela do Reis Carazai, Daniela Ponce, Emanuel Victor Alves Costa, Euler Roberto Fernandes Manenti, Evelin Paola de Almeida Cenci, Frederico Bartolazzi, Glícia Cristina de Castro Madeira, Guilherme Fagundes Nascimento, Isabela Vasconcellos Pires Velloso, Joanna d’Arc Lyra Batista, Júlia Drumond Parreiras de Morais, Juliana da Silva Nogueira Carvalho, Karen Brasil Ruschel, Karina Paula Medeiros Prado Martins, Liege Barella Zandoná, Luanna Silva Monteiro Menezes, Luciane Kopittke, Luís César de Castro, Luiz Antônio Nasi, Maiara Anschau Floriani, Maíra Dias Souza, Marcelo Carneiro, Maria Aparecida Camargos Bicalho, Maria Clara Pontello Barbosa Lima, Mariana Frizzo de Godoy, Milton Henriques Guimarães-Júnior, Paulo Mascarenhas Mendes, Polianna Delfino-Pereira, Raquel Jaqueline Eder Ribeiro, Renan Goulart Finger, Rochele Mosmann Menezes, Saionara Cristina Francisco, Silvia Ferreira Araújo, Talita Fischer Oliveira, Thainara Conceição de Oliveira, Carisi Anne Polanczyk, Milena Soriano Marcolino

**Affiliations:** 1 Brasil Hospital das Clínicas - Universidade Federal de Minas Gerais,Belo Horizonte, MG – Brasil; 2 Brasil Universidade Federal do Rio Grande do Norte, Natal, RN – Brasil; 3 Brasil Faculdade Ciências Médicas de Minas Gerais, Belo Horizonte, MG – Brasil; 4 Brasil Hospital das Clínicas da Universidade Federal de Pernambuco, Recife, PE – Brasil; 5 Brasil Hospital Universitário de Santa Maria,Santa Maria, RS – Brasil; 6 Brasil Hospital São João de Deus,Divinópolis, MG – Brasil; 7 Brasil Hospital Risoleta Tolentino Neves,Belo Horizonte, MG – Brasil; 8 Brasil Hospital Santo Antônio,Curvelo, MG – Brasil; 9 Brasil Hospital Santa Rosália,Teófilo Otoni, MG – Brasil; 10 Brasil Universidade Federal dos Vales do Jequitinhonha e Mucuri, Teófilo Otoni, MG – Brasil; 11 Brasil Hospital UNIMED BH,Belo Horizonte, MG – Brasil; 12 Brasil Hospital Nossa Senhora da Conceição, Cristo Redentor, Porto Alegre, RS – Brasil; 13 Brasil Universidade Estadual Paulista, Botucatu, SP – Brasil; 14 Brasil Centro Universitário de Belo Horizonte (UniBH),Belo Horizonte, MG – Brasil; 15 Brasil Hospital Mãe de Deus,Porto Alegre, RS – Brasil; 16 Brasil Hospital Universitário Canoas,Canoas, RS – Brasil; 17 Brasil Hospitais da Rede Mater Dei,Belo Horizonte, MG – Brasil; 18 Brasil Universidade Federal da Fronteira Sul, Chapecó, SC – Brasil; 19 Brasil Hospital Regional do Oeste, Chapecó, SC – Brasil; 20 Brasil Hospital Universitário Ciências Médicas,Belo Horizonte, MG – Brasil; 21 Brasil Instituto de Avaliação de Tecnologia em Saúde (IATS/ CNPq),Porto Alegre, RS – Brasil; 22 Brasil Hospital Bruno Born, Lajeado, RS – Brasil; 23 Brasil Hospital Luxemburgo, Belo Horizonte, MG – Brasil; 24 Brasil Hospital Municipal Odilon Behrens,Belo Horizonte, MG – Brasil; 25 Brasil Hospital Moinhos de Vento, Porto Alegre, RS – Brasil; 26 Brasil Hospital Santa Cruz,Santa Cruz do Sul, RS – Brasil; 27 Brasil Hospital Regional Antônio Dias,Patos de Minas, MG – Brasil; 28 Brasil Universidade Federal de Ouro Preto, Ouro Preto, MG – Brasil; 29 Brasil Hospital São Lucas (PUCRS),Porto Alegre, RS – Brasil; 30 Brasil Hospital Márcio Cunha, Ipatinga, MG – Brasil; 31 Brasil Hospital Metropolitano Doutor Célio de Castro,Belo Horizonte, MG – Brasil; 32 Brasil Hospital Semper,Belo Horizonte, MG – Brasil

**Keywords:** COVID-19, Coronavirus Infections, Troponin, Artificial Respiration, Mortality

## Abstract

**Background:**

Cardiovascular complications of COVID-19 are important aspects of the disease’s pathogenesis and prognosis. Evidence on the prognostic role of troponin and myocardial injury in Latin American hospitalized COVID-19 patients is still scarce.

**Objectives:**

To evaluate myocardial injury as independent predictor of in-hospital mortality and invasive mechanical ventilation support in hospitalized patients, from the Brazilian COVID-19 Registry.

**Methods:**

This cohort study is a substudy of the Brazilian COVID-19 Registry, conducted in 31 Brazilian hospitals of 17 cities, March-September 2020. Primary outcomes included in-hospital mortality and invasive mechanical ventilation support. Models for the primary outcomes were estimated by Poisson regression with robust variance, with statistical significance of p<0.05.

**Results:**

Of 2,925 patients (median age of 60 years [48-71], 57.1% men), 27.3% presented myocardial injury. The proportion of patients with comorbidities was higher among patients with cardiac injury (median 2 [1-2] vs. 1 [0-2]). Patients with myocardial injury had higher median levels of brain natriuretic peptide, lactate dehydrogenase, creatine phosphokinase, N-terminal pro-brain natriuretic peptide, and C-reactive protein than patients without myocardial injury. As independent predictors, C-reactive protein and platelet counts were related to the risk of death, and neutrophils and platelet counts were related to the risk of invasive mechanical ventilation support. Patients with high troponin levels presented a higher risk of death (RR 2.03, 95% CI 1.60-2.58) and invasive mechanical ventilation support (RR 1.87, 95% CI 1.57-2.23), when compared to those with normal troponin levels.

**Conclusion:**

Cardiac injury was an independent predictor of in-hospital mortality and the need for invasive mechanical ventilation support in hospitalized COVID-19 patients.

## Introduction

Cardiovascular complications of the coronavirus disease 19 (COVID-19)^1 , 2^represent an important aspect of the disease’s pathogenesis and prognosis. Myocardial injury is common in hospitalized patients with COVID-19 and has been reported in 7.2% to 36% of all patients.^3 - 6^This disease has proven to be associated with a poorer prognosis;^7^however, evidence concerning its prognostic role in hospitalized COVID-19 patients in Latin America is still scarce.

In Brazil, many people are still affected by Chagas heart disease and rheumatic valvulopathy.^8^Furthermore, numerous polymorphisms, heterogeneity, and miscegenation exist among the population, which may influence rates of myocardial injury and levels of biomarkers in COVID-19 patients.^9^In a previous analysis performed by our research group, we developed and validated a score with high discriminatory ability to predict mortality among Brazilian patients using data that are easily available on hospital admission, the ABC_2_-SPH score.^10^

The present study aimed to evaluate myocardial injury as an independent predictor of in-hospital mortality and invasive mechanical ventilation support in COVID-19 hospitalized patients, from the Brazilian COVID-19 Registry.

## Materials and methods

This multicenter retrospective cohort study is a substudy of the Brazilian COVID-19 Registry, conducted in 31 Brazilian hospitals in 17 cities from five states (Minas Gerais, Pernambuco, Rio Grande do Sul, Santa Catarina, and São Paulo), detailed in a previous report.^11^The study was approved by the Brazilian National Ethics Committee in Research (CAAE: 30350820.5.1001.0008). Individual informed consent was waived due to the severity of the situation imposed by the pandemic and to the retrospective nature of the study.

This study included consecutive adult patients (aged ≥18 years) with laboratory-confirmed COVID-19,^12^who were admitted to the participating hospitals between March and September 2020 and who had at least one troponin value. Patients with an underlying diagnosis of chronic heart failure (HF) in the medical records, with glomerular filtration rate (GFR) of lower than 30 mL/min/1.73m^2^(estimated by the Chronic Kidney Disease Epidemiology Collaboration [CKD-EPI], set forth by Levey et al.^13^), those who were discharged in less than 24 hours, and those diagnosed with COVID during hospitalization were excluded from the study. Sample size calculation was not performed, all eligible patients were included.

### Data collection

Demographic and clinical characteristics, exams (laboratory, electrocardiogram, and echocardiogram), treatments, and outcome data were collected by trained healthcare professionals and interns in each health center, using the Research Electronic Data Capture (REDCap) tool (version 7.3.1),^14 - 16^at the Telehealth Center of the Federal University of Minas Gerais General Hospital. Comprehensive checks were undertaken to ensure a high-quality data collection. A code was developed in R software to identify non-conforming and inconsistency values, as previously described, based on expert-guided rules, and each study center was contacted to check and correct data, if needed.^10^

### Myocardial injury, troponin assay and study groups

Myocardial injury was defined as an elevation of cardiac troponin (cTnI or cTnT) above the upper reference limit (URL) of the 99^th^percentile, according to the Fourth Universal Definition of Myocardial Infarction.^17^Troponin was measured at the discretion of the treating physician. Troponin values at hospital admission, as well as minimum and maximum values after 24 hours of hospitalization were collected. Any abnormal value among these measures was considered for analysis in the present study. The reference group was composed of patients with troponin values within the normal range.

Troponin assay varied among different centers. Due to the shortage of crucial chemicals at the beginning of the pandemic, some centers used more than one assay during the study period. Therefore, the analysis considered the URL of the 99^th^percentile for each test for men and women, as reported in the Supplemental Material ( Table S1 ).

### Outcomes

The primary outcomes were in-hospital mortality and invasive mechanical ventilation support. Secondary outcomes included cardiovascular complications (acute HF, acute myocardial infarction, and myocarditis), bleeding, thromboembolic events, septic shock, disseminated intravascular coagulation, nosocomial infection, admission to the intensive care unit (ICU), ICU length of stay, hospital length of stay, and need for renal replacement therapy.

### Statistical analysis

Statistical analyses were performed in three main steps: (i) descriptive, (ii) bivariate (evaluation of the association of the outcome with each variable of interest), and (iii) multivariate analyses.

Descriptive analyses were run to describe all variables, stratified into control and study groups (patients with myocardial injury). Categorical variables were described as absolute and relative frequencies. The Shapiro-Wilk normality test was performed to determine whether the continuous variables were normally distributed. As all variables were found to have a non-normal distribution, they were described as medians and interquartile ranges (IQR). The number of comorbidities was defined based on eight comorbidities that had been proved to have a prognostic impact on COVID-19 (hypertension, diabetes mellitus, obesity, coronary artery disease, atrial or flutter fibrillation, cirrhosis, cancer, and previous stroke).^10^

In the bivariate analysis, demographic and clinical variables, and outcomes were assessed using the Fisher’s exact test or the chi-square test to compare proportions, as appropriate. The Kruskal-Wallis test was used to compare medians of continuous variables, while the Dunn´s test was used as a post-hoc test. For the multivariate analyses, two predictive models were estimated to evaluate the role of elevated troponin on the primary outcomes: in-hospital mortality and invasive mechanical ventilation support. All variables included in the models were obtained at hospital admission. A set of potential predictive variables for the primary outcomes was selected *a priori* (supplemental material - Figure S3 ) based on previous scientific evidence of variables associated with a worse prognosis of COVID-19.^10^Laboratory tests were performed at the discretion of the attending physician. Imaging test results were not included, since they are not always performed on hospital admission, and their interpretation involves the examiner’s judgment.

Models for the primary outcomes were estimated by Poisson regression with robust variance. In the model to predict invasive mechanical ventilation support, patients who were on invasive mechanical ventilation at admission were not included (n=72). Poisson regression was chosen due to the ability to estimate the relative risk (RR), which is the parameter of primary interest, since an elevated event rate was expected.^18 , 19^

The shaping of the prediction models divided the variables into five blocks by a stepwise-forward approach,^20^mutually inserted in the regression models one to five. As the main goal of the analysis was to identify the association of myocardial injury with the study outcomes, this variable was tested in all five models. The first one included only myocardial injury. The second added age and sex, the third added the number of comorbidities, and the fourth added clinical characteristics on hospital admission. The fifth multivariate model contained only the variables with a 5% significance level after adjusting for the other variables added to the previous multivariate models. The variables were included from the largest to the least significance, to test which associations between the explicative variables and the outcomes would remain significant throughout the process.

The statistical significance of the variables that were part of the models was evaluated by analyzing the RR and their respective 95% confidence intervals (95% CI), as well as by the p-value of the tests, aimed at reducing the probability of type I error. A comparison of the models’ goodness-of-fit tests was performed using the Akaike Information Criterion.^20^

For the regression models, the RR and their respective 95% CI were estimated. All analyses were performed in the STATA software (StataCorp. 2012. Stata Statistical, version 12) and R software (version 4.0.2), using the tidyverse, lubridate, stringi, rlang, jsonlite, Rcurl, writexl, openxlsx, readxl, and sandwich packages. A p-value <0.05 was considered statistically significant.

## Results

### Baseline characteristics

Of 7,760 patients, 2,925 were included in the present analyses ( Figure 1 ). Demographic and clinical characteristics are shown in Table 1 . Patients with myocardial injury had a median age of 10 years older than the controls, a higher number of comorbidities, and a higher prevalence of underlying hypertension, coronary artery disease, ischemic stroke, atrial fibrillation, diabetes, chronic obstructive pulmonary disease (COPD), cancer, and chronic kidney disease (CKD). Additionally, there was a higher proportion of individuals with myocardial injury with abnormal mental state and lower peripheral oxygen saturation (SpO_2_)/fraction of inspired oxygen (FiO_2_) ratio (SF ratio) at hospital admission. Regarding laboratory parameters, patients with myocardial injury had higher median levels of C-reactive protein (CRP), lactate dehydrogenase (LDH), and N-terminal pro-brain natriuretic peptide (NT-proBNP)/brain natriuretic peptide (BNP), when compared to the controls. Electrocardiogram and echocardiogram results are reported in the supplemental material ( Table S2 ).

**Figure 1:** – Flowchart of patients included in the study.

**Table 1:** – Demographic and clinical characteristics of the study population upon hospital admission

Variables	Study group (n = 832) N (%)	Controls (n = 2,093) N (%)	Total (n = 2,925) N (%)	p-value
Men	456 (27.3%)	1213 (72.7%)	1669 (57.1%)	0.121
Age ( *median* )	67 (57-77)	57 (45-67)	60 (48-71)	0.000
**Comorbidities**				
Overall number ( *median* )	2 (1-2)	1 (0-2)	1 (0-2)	0.000
**Cardiovascular diseases**				
Hypertension	533 (64.1%)	1.010 (48.3%)	1.543 (52.7%)	0.000
Coronary artery disease	62 (7.5%)	82 (3.9%)	144 (4.9%)	0.000
Ischemic stroke	42 (5.1%)	40 (1.9%)	82 (2.8%)	0.000
*Atrial fibrillation/flutter*	38 (4.6%)	26 (1.2%)	64 (2.2%)	0.000
Chagas disease	2 (0.2%)	2 (0.1%)	4 (0.1%)	0.321
Rheumatic valve disease	2 (0.2%)	0 (0.0%)	2 (0.1%)	0.081
**Metabolic diseases**				
Diabetes mellitus	289 (34.7%)	528 (25.2%)	817 (27.9%)	0.000
Obesity	167 (20.1%)	425 (20.3%)	592 (20.2%)	0.887
**Respiratory diseases**				
Asthma	149 (5.9%)	151 (7.2%)	200 (6.8%)	0.200
COPD	75 (9.0%)	95 (4.5%)	170 (5.8%)	0.000
**Other conditions**				
Cancer	62 (7.5%)	78 (3.7%)	140 (4.8%)	0.000
Rheumatic disease	20 (2.4%)	32 (1.5%)	52 (1.8%)	0.106
Chronic renal disease	25 (3.0%)	14 (0.7%)	39 (1.3%)	0.000
HIV	5 (0.6%)	13 (0.6%)	18 (0.6%)	0.950
Cirrhosis	7 (0.8%)	4 (0.2%)	11 (0.4%)	0.016
**Clinical characteristics**	**(n = 832)**	**(n = 2.093)**	**(n = 2.925)**	
*Glasgow <15*	170 (20.4%)	156 (7.5%)	326 (11.1%)	0.000
	**(n = 810)**	**(n = 2.057)**	**(n = 2.867)**	
*SF ratio (median)*	362 (213-438)	433 (343-457)	424 (329-452)	0.000
**Systolic blood pressure**	**(n = 787)**	**(n = 1995)**	**(n = 2782)**	
≥90 (mmHg)	770 (97.8%)	1.997 (99.1%)	2.747 (98.7%)	0.003
<90 (mmHg)	9 (1.1%)	15 (0.7%)	24 (0.9%)	
Inotrope requirement	8 (1.0%)	3 (0.1%)	11 (0.4%)	
**Diastolic blood pressure**	**(n = 796)**	**(n = 1989)**	**(n = 2785)**	
> 60 (mmHg)	620 (77.9%)	1.737 (87.3%)	2.357 (84.6%)	0.000
≤ 60 (mmHg)	100 (12.6%)	210 (10.6%)	310 (11.1%)	
Inotrope requirement	76 (9.5%)	42 (2.1%)	118 (4.2%)	

*COPD: chronic obstructive pulmonary disease; HIV: human immunodeficiency virus; SF ratio: peripheral oxygen saturation (SpO_*2*_)/fraction of inspired oxygen (FiO_*2*_) ratio.*

Patients with myocardial injury had higher in-hospital mortality and a higher frequency of all secondary outcomes ( Table 2 ). Multivariate regression models for in-hospital mortality and invasive mechanical ventilation support have shown that myocardial injury was a significant predictor for both outcomes, even when adjusting for the other variables. A decrease was found in the impact of elevated troponin levels on the multivariate model for in-hospital mortality ( Table 3 ), with the addition of the variables to the model. In the fifth multivariate model, patients with elevated troponin levels presented a higher risk of death when compared to the controls (RR: 2.03 [1.60-2.58]). Age, number of comorbidities, respiratory rate, SF ratio, and CRP on admission were also associated with a higher risk of death. Myocardial injury also proved to be an independent predictor of invasive mechanical ventilation support (RR: 1.87 [1.57-2.23]) ( Table 4 ). The number of comorbidities, respiratory rate, CRP, and number of neutrophils were associated with an increased risk of invasive mechanical ventilation support. By contrast, high SF ratio and platelet count were associated with a reduced risk of invasive mechanical ventilation support. For information on models, see supplemental material S4. The central illustration of the main results of the article.

**Table 2:** – Clinical outcomes of study patients

Variables	Study group (n = 832) N (%)	Controls (n = 2,093) N (%)	Total (n = 2,925) N (%)	p-value
**Clinical assessment**				
Hospital length of stay ( *median* )	14 (7-25)	7 (4-13)	9 (5-16)	0.000
Admission to the ICU	584 (70.2%)	765 (36.6%)	1.349 (46.1%)	0.000
Length of stay in the ICU (days) ( *median* )	12 (6-22)	8 (4-16)	10 (5-19)	0.000
Invasive mechanical ventilation	509 (61.3%)	467 (22.3%)	976 (33.4%)	0.000
Acute respiratory distress syndrome	325 (39.1%)	428 (25.5%)	753 (25.7%)	0.000
Septic shock	258 (31.0%)	201 (9.6%)	459 (15.7%)	0.000
Nosocomial infection	186 (22.4%)	189 (9.0%)	375 (12.8%)	0.000
Hyperglycemia	148 (17.8%)	215 (10.3%)	363 (12.4%)	0.000
**Vascular thrombosis**	81 (9.7%)	125 (6.0%)	206 (7.1%)	0.000
Pulmonary thromboembolism	57 (6.9%)	95 (4.5%)	152 (5.2%)	0.011
Deep vein thrombosis	25 (3.0%)	31 (1.5%)	56 (1.9%)	0.007
Arterial thrombosis	6 (0.7%)	4 (0.2%)	10 (0.3%)	0.027
Acute or decompensated heart failure	33 (4.0%)	23 (1.1%)	56 (1.9%)	0.000
Acute myocardial infarction	25 (3.0%)	2 (0.1%)	27 (0.9%)	0.000
Myocarditis	7 (0.8%)	2 (0.1%)	9 (0.3%)	0.001
Bleeding	26 (53.1%)	24 (46.9%)	49 (1.7%)	0.000
Disseminated intravascular coagulation	7 (0.8%)	6 (0.3%)	13 (0.4%)	0.042
Death	376 (45.2%)	218 (10.4%)	594 (20.3%)	0.000

*ICU: intensive care unit.*

**Table 3:** – Predictors (at hospital admission) of in-hospital mortality by Poisson regression model with robust variance

Variables	Multivariate model 1 RR (95% CI)	Multivariate model 2 RR (95% CI)	Multivariate model 3 RR (95% CI)	Multivariate model 4 RR (95% CI)	Multivariate model 5 RR (95% CI)
Myocardial injury	4.2482 (3.2820-5.4982)*	3.1956 (2.4335- 4.1962)*	3.1077 (2.4364- 3.9640)*	1.9057 (1.4843-2.4468)*	2.0323 (1.5995-2.5822)*
Age	---	1.0285 (1.0191-1.0379)*	1.0273 (1.0190- 1.0357)*	1.0262 (1.0176-1.0349)*	1.0269 (1.0192-1.0348)*
Female sex	---	0.8122 (0.6289-1.0489)***	0.7924 (0.6461-0.9718)**	0.9553 (0.7736-1.2621)***	---
Number of comorbidities****	---	---	1.1550 (1.0317- 1.2930)**	1.1089 (1.0187-1.2070)*	8.1862 (7.5153-8.9168)*
Respiratory rate	---	---	---	1.0213 (1.0018-1.0429)**	1.0223 (1.0086-1.0362)**
Heart rate	---	---	---	1.0039 (0.9994-1.0083)***	---
Systolic blood pressure <90 mmHg without inotropes	---	---	---	0.2508 (0.1062-0.5920)**	---
Systolic blood pressure <90 mmHg with inotropes	---	---	---	1.3022 (0.7688-2.2056)***	---
Glasgow <15	---	---	---	1.0723 (0.7372-1.5599)***	---
SF ratio	---	---	---	0.9971 (0.9959-0.9983)*	0.9969 (0.9963-0.9977)*
Invasive mechanical ventilation	---	---	---	0.9714 (0.5843-1.6148)***	---
C-reactive protein	---	---	---	1.0010 (1.0006-1.0027)*	1.0020 (1.0009-1.0026)*
Hemoglobin	---	---	---	1.0150 (0.9644-1.0682)***	---
Neutrophils	---	---	---	1.0000 (1.0000-1.0000)*	---
Platelet count	---	---	---	0.9994 (0.9993-0.9996)*	0.9994 (0.9993-0.9999)*
Urea	---	---	---	1.0046 (1.0001-1.0103)**	---
Lactate	---	---	---	0.9775 (0.9421-1.0143)***	---
Sodium	---	---	---	0.9948 (0.9763-1.0136)***	---
Bicarbonate	---	---	---	0.9716 (0.9443-0.9996)*	---
pH	---	---	---	0.9987 (0.9982-0.9993)*	---
pCO_2_	---	---	---	1.0034 (0.9939-1.0129)***	---
D-dimer	---	---	---	0.9999 (0.9998-1.0001)***	---

CI: confidence interval; RR: relative risk; SF ratio: peripheral oxygen saturation (SpO_*2*_)/fraction of inspired oxygen (FiO_*2*_) ratio. *p<0.0001; **p<0.05; ***p>0.05; ****Hypertension, diabetes mellitus, obesity, coronary artery disease, heart failure, atrial fibrillation or flutter, cirrhosis, cancer, and previous stroke.

**Table 4:** – Predictors (at hospital admission) of invasive mechanical ventilation support by Poisson regression model with robust variance

Variables	Multivariate model 1 RR (95% CI)	Multivariate model 2 RR (95% CI)	Multivariate model 3 RR (95% CI)	Multivariate model 4 RR (95% CI)	Multivariate model 5 RR (95% CI)
Myocardial injury	2.8607 (2.4221-3.3787)*	2.6906 (2.1353- 3.3901)*	2.5901 (2.1627-3.1021)*	1.9018 (1.5842-2.2830)*	1.8675 (1.5662-2.2268)*
Age	---	1.0057 (0.9983-1.0131)***	1.0034 (0.9976-1.0093)***	1.0068 (1.0002-1.0133)*	---
Female sex	---	0.8889 (0.7492-1.0546)***	0.8697 (0.7492-1.0546)***	0.9398 (0.7852-1.1249)***	---
Overall comorbidities****	---	---	1.1781 (1.0936-1.2691)*	1.1313 (1.0494-1.2196)**	1.1295 (1.0488-1.2164)*
Respiratory rate	---	---	---	1.0369 (1.0254-1.0485)*	1.0332 (1.0222-1.0443)*
Heart rate	---	---	---	1.0002 (0.9954-1.0051)***	---
Systolic blood pressure < 90 mmHg with no inotrope	---	---	---	0.4001 (0.1252-1.2781)***	---
Glasgow <15	---	---	---	0.8320 (0.6109-1.1332)***	---
SF ratio	---	---	---	0.9980 (0.9960-0.9990)*	0.9980 (0.9970-0.9990)*
C-reactive protein	---	---	---	1.0027 (1.0019-1.0034)*	1.0030 (1.0020-1.0040)*
Hemoglobin	---	---	---	1.0111 (0.9641-1.0605)***	---
Neutrophils	---	---	---	1.0005 (1.0003-1.0006)*	1.0004 (1.0002-1.0005)*
Platelet count	---	---	---	0.9980 (0.9970-0.9990)*	0.9995 (0.9994-0.9997)*
Urea	---	---	---	0.9954 (0.9912-0.9997)**	---
Lactate	---	---	---	0.9740 (0.9045-1.0487)***	---
Sodium	---	---	---	0.9869 (0.9688-1.0054)***	---
Bicarbonate	---	---	---	1.0439 (0.9752-1.1174)***	---
pH	---	---	---	0.0965 (0.0039-2.3727)***	---
pCO_2_	---	---	---	0.9743 (0.9413-1.0084)***	---
D-dimer	---	---	---	0.9999 (0.9998-1.0000)***	---

CI: confidence interval; RR: relative risk; SF ratio: peripheral oxygen saturation (SpO_*2*_)/fraction of inspired oxygen (FiO_*2*_) ratio. *p<0.0001; **p<0.05; ***p>0.05; ****Hypertension, diabetes mellitus, obesity, coronary artery disease, heart failure, atrial fibrillation or flutter, cirrhosis, cancer, and previous stroke.

**Central Illustration:** : Myocardial Injury and Prognosis in Hospitalized COVID-19 Patients in Brazil: Results From The Brazilian COVID-19 Registry

Central illustration of the main results. BNP: brain natriuretic peptide; CRP: C-reactive protein; ICU: intensive care unit; LDH: lactate dehydrogenase; NT-proBNP: N-terminal pro-brain natriuretic peptide.

## Discussion

In this multicenter cohort study, with a large sample of 31 Brazilian hospitals, patients with myocardial injury were 10 years older (median) than the control, and had a higher prevalence of underlying comorbidities, worse clinical parameters at hospital admission, and higher levels of CRP, LDH, and NT-proBNP/BNP when compared to those without myocardial injury. Myocardial injury was an independent predictor of in-hospital mortality (RR: 2.03 [1.60-2.58]) and invasive mechanical ventilation support (RR: 1.87 [1.57-2.23]). Higher number of comorbidities, higher respiratory rate, and higher levels of CRP were associated with a higher risk of in-hospital mortality and invasive mechanical ventilation support. Additionally, age and lower SF ratio were also independently associated with in-hospital mortality, while the number of neutrophils was associated with an increased risk of invasive mechanical ventilation support. Patients with myocardial injury showed a higher frequency of cardiovascular complications, bleeding, thromboembolic events, septic shock, disseminated intravascular coagulation, nosocomial infection, admission to the ICU, ICU and hospital length of stay, and need for renal replacement therapy.

The present study’s findings are in line with results of studies from other countries, which reported remarkably similar characteristics of COVID-19 patients who developed myocardial injury, including advanced age and a high prevalence of underlying medical conditions.^6 , 21 - 23^There was no evidence of sex differences in the prevalence of the variables analyzed among patients with myocardial injury, which contrasts with data previously published in the literature. In the systematic review performed by Toraih et al.,^24^including 17,794 patients with cardiac injury assessed by troponin measurement, the authors found a significantly higher proportion of men with critical illnesses, defined as acute respiratory distress syndrome, invasive mechanical ventilation, and ICU admission. In the present analysis, older women with myocardial injury showed a higher risk for death and the need for invasive mechanical ventilation, but with no statistical significance in the latter.

In fact, troponin and other biomarkers are commonly abnormal in patients hospitalized with COVID‐19. There are different mechanisms for the development of cardiac damage in COVID-19 patients, such as increased cardiac effort in acute respiratory failure,^25^and SARS-CoV-2 interaction with angiotensin-converting enzyme 2 receptors.^2 , 26 - 28^Although the specific mechanisms of myocardial injury are uncertain, the mechanisms proposed include inflammatory response and immune system disorders during disease progression.^26 , 29^

Results of the tests used to assess cardiac function and injury have shown significantly higher values in COVID-19 patients who died than those who were discharged alive. Our results are in line with these findings, as BNP, creatine phosphokinase, LDH, NT-proBNP, and CRP, which is a biomarker of inflammatory response, were higher in patients with myocardial injury. A recent study which included 187 COVID-19 patients hospitalized in Rio de Janeiro has also reported troponin as an independent predictor for adverse events. Meanwhile, BNP was not an independent predictor for mortality or the need for invasive mechanical ventilation support.^29^This finding may be limited by the small sample size and high number of missing values.

A systematic review and meta-analysis of Chinese cohort studies by Alzahrani and Al-Rabia^2^showed that 45.2% of the patients with COVID-19-induced myocardial injury have died.^26^In the present study, there was a 4.25-fold higher mortality rate in patients with myocardial injury when compared to the controls, as well as a higher median hospital length of stay, ICU admissions, and all other aforementioned secondary outcomes. Hence, COVID-19 patients with cardiac injury were more susceptible to disease complications and had poorer prognoses. These findings are consistent with results reported by studies performed in other countries.^6 , 31 , 32^

Currently, several studies have proposed clinical variables, laboratory and chest X-ray findings for the prediction of the risk of severe COVID-19 progression and mortality.^33^In a previous analysis, our research group developed and validated a risk prediction score for in-hospital mortality in COVID-19 patients (available through the link https://abc2sph.com/pt/), which includes older age, blood urea nitrogen, number of comorbidities, CRP, peripheral SF ratio, platelet count, and heart rate as predictors. However, troponin was not included in those analyses.^10^In the present study, cardiac injury was an independent risk factor for mortality and invasive mechanical ventilation support, in addition to the variables previously tested, which was the most important finding of the current analyses. These results play an important role in patient care, as they can help healthcare professionals identify patients who may have a worse prognosis, guiding interventions for the management of clinical conditions and improvements in health care. A logical next step would be to assess the inclusion of troponin to the ABC_2_-SPH risk score, an important topic for future studies concerning ABC_2_-SPH. It is our hope that this cohort can also be useful in further studies to create a predictive score for myocardial injury.

This study has limitations. First, we cannot assure national representativeness of participating hospitals. Since this is a multicentric analysis of COVID-19, troponin tests could have inconsistencies, given that multiple commercial laboratory kits were used in an inter- and intra-institutional manner, and different reference values were standardized in each study, leading to measurement bias. Another limitation was that the grouped analyses did not allow the recognition of the contribution of each center to the outcomes of death and invasive mechanical ventilation support, as well as institutional factors related to mortality.^34^It is important to mention that the pandemic period when the data were collected involved a population that had not been vaccinated yet,^35^and part of the patients in the study group might not have developed myocardial injury, but rather acute myocardial infarction. Due to the potential risk of transmissibility of COVID-19, there is a possibility that no differential diagnostic technique is performed in some cases.^36^Furthermore, since the present study was an observational study, other variables that may be confounding and unmeasurable or unrecognized may not have been collected or analyzed.

Regarding the study’s strengths, a strict methodological criterion was used to perform this study, which was based on a robust patient sample, with a confirmed COVID-19 diagnosis. The sample was obtained by the collaboration of researchers from 31 public, private, and mixed hospitals of different sizes and complexity levels, from different Brazilian regions, in order to guarantee a diversity of the studied population. However, they are not representative of the entire healthcare system of the country.

## Conclusion

Cardiac injury, measured by elevated troponin levels, was an independent predictor of mortality and invasive mechanical ventilation support among hospitalized COVID-19 patients, as were the variables of a recently validated risk prediction score. Future strategies involving the frequent monitoring of troponin levels as risk biomarkers in patients with COVID-19 during their hospitalization should be tested to investigate their role in reducing the risk of complications and death, as well as in improving patient care.

## * Supplemental Materials

For additional information, please, click here
